# St. John’s Wort-Induced Supraventricular Tachycardia

**DOI:** 10.7759/cureus.14356

**Published:** 2021-04-07

**Authors:** Kristy A Fisher, Poonam Patel, Samer Abualula, Livasky Concepion

**Affiliations:** 1 Psychiatry, Aventura Hospital and Medical Center, Aventura, USA; 2 Psychiatry, Nova Southeastern University Dr. Kiran C. Patel College of Osteopathic Medicine, Davie, USA; 3 Internal Medicine, Aventura Hospital and Medical Center, Aventura, USA

**Keywords:** cyp450, svt, internal medicine, consultation liaison psychiatry, st. john’s wort, supraventricular tachycardia, hypericum perforatum, depression, anxiety, palpitations

## Abstract

St. John’s wort, a non-FDA-approved over-the-counter (OTC) herbal supplement with antidepressant activity known as *Hypericum perforatum*, has been found to induce supraventricular tachycardia (SVT) in the absence of any underlying structural cardiac abnormality or known medical history via currently unclear pathophysiology. In this case presentation, the authors present a case of a 33-year-old female who presented with recurrent episodes of palpitations one month after initiating St. John’s wort for the treatment of depression, which ultimately resolved upon cessation of the herbal supplement. Therefore, the postulated insinuating event is suspected to be the ingestion of St. John’s wort. This will be the first documented case of St. John’s wort-induced SVT. This case study brings into question the safety of the use of this agent as an OTC supplement for the management of depression. Further investigation is required to aid in the knowledge and understanding of the causative mechanism and to identify those patients who are at potentially heightened risks of such manifestations.

## Introduction

St. John’s wort in the management of depression

*Hypericum perforatum* has been widely used for centuries throughout traditional medicine as an over-the-counter (OTC) agent to treat depression due to its efficacy in mild to moderate depression, along with its relatively safe side-effect profile [1], which includes hepatotoxicity, cardiovascular compromise, gastrointestinal upset, induction of mania, dizziness, confusion, fatigue, anxiety, and photosensitivity, along with major drug interactions due to induction of the CYP 3A4 and 2Cp enzymes of the cytochrome P450 system [1, 2]. The active principle accountable for the antidepressant effects is unknown, but suspected agents include hypericin, pseudohypericin, and hyperforin [2]. The mechanism of action responsible for the antidepressant properties of St. John’s wort includes inhibition of the serotonin, norepinephrine, and dopamine reuptake, activation of transient receptor potential C6 channels with possible influence of monoamine uptake (specifically hyperforin), stimulation of the development and function of oligodendrocytes, and suppression of voltage-dependent calcium channel and mitogen-activated protein kinase resulting in release of glutamate (specifically hypericin) [3-7]. 

Supraventricular tachycardia

Supraventricular tachycardia (SVT), a broad term encompassing many heart arrhythmias, is an abnormally fast heartbeat that arises in the atrial tissue or atrioventricular node (AV) node. One example, and the most common SVT (excluding atrial fibrillation and atrial flutter), is AV-nodal reentrant tachycardia (AVNRT). Causation is via reentrant conduction within the AV node, utilizing both the fast and the slow pathways. Electrocardiogram (ECG) usually indicates the absence of a P wave preceding the QRS complex; the P wave can appear as a pseudo R’ wave in lead V1 and pseudo S’ in the inferior leads. Acute termination is via physical maneuvers (vagal stimulus) or adenosine. Treatment preventing recurrent AVNRT includes AV nodal blocking therapy with B blockers or nondihydropyridine calcium channel blockers. If frequent AVNRT continues or the patient prefers to avoid long-term medical therapy, catheter ablation is warranted, which carries a 1% risk of AV nodal injury requiring pacemaker implantation [8].

## Case presentation

The patient is a 33-year-old female with a reported history of depression for a duration of one month, who presented with recurrent episodes of palpitations after a night of salsa dancing. The patient reported recent feelings of depression attributed to marriage issues. The patient began seeing a psychotherapist specialized in marriage counseling three weeks prior to presentation, who recommended the use of St. John’s wort at a dose of 300 mg daily. Three weeks post-initiation of pharmacologic treatment, the patient reported sweating, insomnia, and frequent episodes of palpitations both at rest and with exertion, which exhibited persistence of less than one minute with spontaneous resolution.

At presentation, the patient was awake, alert, and oriented, with a palpable carotid pulse and heart rate (HR) of 150-160. The patient denied chest pain, shortness of breath, dizziness, or presyncopal symptoms. On site, a Valsalva and carotid artery massage was performed simultaneously, with resultant acute abruption of tachycardia. Upon arrival to the emergency department (ED), another episode occurred with similar presentation (HR: 150-160 bpm; blood pressure (BP): 110/68 mmHg; oxygen saturation and respiratory rate within normal limits; denied chest pain, shortness of breath, or presyncopal symptoms). ECG revealed SVT with HR 148 bpm, with no preceding P wave, pseudo R’ on V1, and pseudo deep S’ in the inferior leads. 

Physical exam was deemed unremarkable, aside from tachycardia and reported anxiety, which the patient attributed to the palpitations. All laboratory findings were within normal limits, including complete blood count (CBC) and comprehensive metabolic panel (CMP), with negative toxicology screen, undetectable blood alcohol level, and troponin x1. The patient received lorazepam 1 mg IV. After five minutes of attempted Valsalva maneuver, the rhythm converted to sinus rhythm (SR) without the administration of adenosine. The patient was discharged home from the ED, with a scheduled electrophysiologist (EP) outpatient follow-up.

Upon EP follow-up, repeat electrolytes were normal, with a transthoracic echocardiogram (TTE) revealing normal ejection fraction (EF) at 60-65%, no wall motion abnormality, normal cardiac valves, normal cardiac structures, and dimension with right ventricular systolic pressure (RVSP) 26. Recommendations included immediate discontinuation of St. John’s wort herbal supplement, with strict observation and no medical or invasive interventions deemed necessary. The patient continued to report episodes of palpitations, persisting anywhere from 30 seconds to five minutes, with either resolution spontaneously or with the personal performance of the modified Valsalva maneuver (strain for 15 seconds in the semi-recumbent position followed by supine repositioning with 15 seconds of passive leg raise at 45-degree angle). After three weeks of cessation of St. John’s wort herbal supplement, complete resolution was reported. 

## Discussion

Depression is a widely undiagnosed and untreated medical condition characterized by low mood, anhedonia, and fatigue that can affect a patient’s thoughts, behavior, motivation, feelings, and overall health [9]. Depressed mood is characterized as a symptom contributing to mood disorders, such as major depressive disorder (MDD), dysthymia, and bipolar disorder, and may even manifest as a result of a normal reaction to a life stressor. In general, depression is a major contributor to worldwide disability, with an estimated 350 million people affected [10]. Of those, 43% suffering from severe depression in the USA report having dysfunctions in their daily life [11]. Selective serotonin reuptake inhibitors (SSRIs) are considered the first line in the management of depression. Although not currently US Food and Drug Administration (FDA)-approved in the management of depressive disorders, St. John’s wort as a monotherapy has been found to be superior to placebo [1], with a demonstration of comparable efficacy to SSRIs in the treatment of mild to moderate depression [12]. The chronicity, recurrent nature, and required long-term treatment of depression contribute to the preference of a more holistic or natural agent for its management. This, along with the cost-efficacy, availability, and relatively safe side-effect profile of St. John’s wort, leads patients to prefer it as a treatment over SSRIs in the management of depression [13].

St. John’s wort is currently considered as either a complementary, augmenting, or alternative medicine by many practitioners, including general practitioners (GP) and psychiatrists. However, many are still hesitant to recommend its use due to the limited research with a resultant lack of knowledge. The agent remains “off-label” and oftentimes, due to its easy accessibility over the counter and multiple available formulations, patients do not always disclose its use to their doctor [14]. In a study conducted among psychiatrists, almost half of the participants reported that at least one of their patients regularly used St. John's wort, with 25% of the participants recommending the treatment themselves. Given this shift to modern and “off-label” medicine in the general population, participating physicians reported that their patients should be educated about using St. John's wort and informed of its side effect profile and drug-to-drug interactions [15].

Though St. John's wort has a relatively low side effect profile when used in isolation, the majority of adverse reactions were found to arise from drug-to-drug interactions. Many cases of serotonin syndrome (SS) in patients who are taking St. John's wort along with pharmaceutical antidepressants have been documented [16]. SS is a medication-induced condition resulting from excessive stimulation of the central and peripheral synaptic 5-HT2A serotonin receptors, with subsequent potentially life-threatening consequences due to autonomic and neuromuscular aberrations. Formal diagnostic criteria for SS do not exist currently due to the variety of possibly exhibited symptoms (ranging from mild, flu-like symptoms to severe cardiovascular collapse and death) and the lack of present standard confirmatory testing. Therefore, diagnosis is clinically based on the contingent triad of altered mental status, autonomic hyperactivity, and neuromuscular abnormalities in patients exposed to medications with the propensity to increase the effects of serotonin [17]. The hyperforin extract found in St. John's wort has been shown to modulate the level of neurotransmitters, such as serotonin, by weakening the activity of catechol-O-methyl transferase, thus reducing the uptake of serotonin [16]. However, SS has never been shown with the use of St. John’s wort as a monotherapy in the management of depression.

When treating patients with depression, it is imperative to also consider co-existing psychiatric (most commonly, anxiety disorders) and medical comorbidities, which can lead to difficulty in the proper evaluation/examination, diagnosis, and treatment[18]. Furthermore, psychiatric conditions may mimic medical conditions and vice versa. One issue is the overlapping symptomatology of panic attacks and multiple cardiovascular manifestations. Oftentimes, specialists generally treat the acutely occurring symptoms specific to their specialty before addressing the full range of concerns, making it difficult to establish the underlying causative nature of all presenting symptoms. This is of particular concern with cardiologists and psychiatrists when faced with determining the causative chronology of panic attacks (episodes of palpitations, chest pain, dizziness, light-headedness, difficulty breathing, numbness and tingling in hands, flushes, or chills) vs. paroxysmal supraventricular tachycardia (PVST).

For example, one study discussing the pathophysiology of this shared symptomatology hypothesized that panic disorders may be due to an underlying arrhythmia rather than a primary psychiatric disorder. This postulation manifested from the findings of PVST as the root cause of “panic attacks” rather than the consequence. Supporting evidence included complete cessation of “panic attacks” following catheter ablation in patients previously diagnosed with panic disorder [19]. Though this may provide some insight and direction for practitioners, further investigation is needed in regards to the actual prevalence of SVT as the underlying mechanism of panic attacks in order to properly guide appropriate diagnosis and treatment/management.

## Conclusions

St. John’s wort has the propensity to elicit SVT in a healthy cardiovascular system. The mechanism to which this can occur is currently unknown. Further investigation is required to aid in the knowledge and understanding of the causative mechanism and to identify those patients who are at potentially heightened risks of such occurrences. Prospective studies encompassing a larger patient population in which occurrence of SVTs are comprehensively assessed to provide solidifying evidence and explore a potential pathophysiology of the development of SVT under the influence of St. John’s wort are needed. Furthermore, the information presented in this case study brings into question the safety of the use of St. John’s wort as an OTC supplement used in the management of depression. Potential studies could explore its safety as a monotherapy, as well as its safety with concomitant medications typically used in the management of depression and anxiety, including SSRI’s and benzodiazepines.
